# Ochratoxin A: *In Utero* Exposure in Mice Induces Adducts in Testicular DNA

**DOI:** 10.3390/toxins2061428

**Published:** 2010-06-11

**Authors:** Jamie E. Jennings-Gee, Mariana Tozlovanu, Richard Manderville, Mark Steven Miller, Annie Pfohl-Leszkowicz, Gary G. Schwartz

**Affiliations:** 1 Department of Cancer Biology, Wake Forest University, Winston-Salem, North Carolina, USA; Email: jjenning@wfubmc.edu (J.E.J-G.); msmiller@wfubmc.edu (M.S.M.); 2 Laboratory Chemical engineering, Department Bioprocess & Microbial System, UMR CNRS/INPT/UPS 5503, ENSA Toulouse, France; Email: mariana.tozlovanu@ensat.fr (M.T.); 3 Department of Chemistry, University of Guelph, Guelph Ontario, Canada; Email: rmanderv@uoguelph.ca (R.M.); 4 Department of Cancer Biology, Urology, and Epidemiology and Prevention, Wake Forest University, Winston-Salem, North Carolina, USA

**Keywords:** ochratoxin, testicular cancer, DNA adduct, transplacental contamination, epidemiology

## Abstract

Ochratoxin A (OTA) is a nephrotoxin and carcinogen that is associated with Balkan endemic nephropathy and urinary tract tumors. OTA crosses the placenta and causes adducts in the liver and kidney DNA of newborns. Because the testis and kidney develop from the same embryonic tissue, we reasoned that OTA also may cause adducts transplacentally in the testis. We tested the hypothesis that acute exposure to OTA, via food and via exposure *in utero*, causes adducts in testicular DNA and that these lesions are identical to those that can be produced in the kidney and testis by the consumption of OTA. Adult mice received a single dose of OTA (from 0–1,056 µg/kg) by gavage. Pregnant mice received a single i.p. injection of OTA (2.5 mg/kg) at gestation day 17. DNA adducts were determined by ^32^P-postlabeling. Gavage-fed animals sacrificed after 48 hours accumulated OTA in kidney and testis and showed DNA adducts in kidney and testis. Some OTA metabolites isolated from the tissues were similar in both organs (kidney and testis). The litters of mice exposed prenatally to OTA showed no signs of overt toxicity. However, newborn and 1-month old males had DNA adducts in kidney and testis that were chromatographically similar to DNA adducts observed in the kidney and testis of gavage-fed adults. One adduct was identified previously as C8-dG-OTA adduct by LC MS/MS. No adducts were observed in males from dams not exposed to OTA. Our findings that *in utero* exposure to OTA causes adducts in the testicular DNA of male offspring support a possible role for OTA in testicular cancer.

## 1. Introduction

Ochratoxin A (OTA) is a mycotoxin produced by species of *Aspergillus* and *Penicillium* that has long been studied as a nephrotoxin, immunotoxin, teratogen and carcinogen (for recent reviews see [1,2,3]). OTA contamination of grain is the cause of mycotoxic porcine nephropathy (a.k.a., “Danish nephropathy”), a renal disease of swine that is an important cause of economic losses in the pork industry [4,5] and may be responsible for some cases of nephropathy and cancer in humans [2]. Specifically, OTA is strongly implicated as the cause of Balkan endemic nephropathy (BEN), a fatal tubulointerstitial disease that is associated with renal atrophy [2,6,7] and that exhibits features similar to porcine nephropathy in Bulgaria [5,7]. Approximately 30% of patients dying with BEN have tumors of the urinary tract [6,8]. High amounts of OTA are found in human sera in regions where OTA contamination of food stuffs is high, particularly northern Europe (for reviews see [2,7]). 

Several areas of the Balkans that are known to have high levels of OTA-contamination of food have recently experienced a marked increase in the incidence of testicular cancer. For example, from 1960 to 1985, the incidence of testicular cancer in Vas, Hungary, increased 5-fold, from 1.2 to 6.1/100,000 [9]. A similar increase was reported for Slovenia [9]. A possible etiologic role for OTA in testicular cancer in men was hypothesized in 2002 by Schwartz [10], who noted that the unusual geographic distribution of testis cancer, with especially high rates in Denmark, was correlated with the national *per capita* consumption of OTA-contaminated foods. Recent toxicologic data in rodents support the concept that dietary exposure to OTA can influence carcinogenesis in the testis. For example, Ueta *et al.*, [11] administered 2 mg/kg OTA intraperitoneally to Pdn/Pdn mice on day 7.5 of gestation. They observed a significant depression of *Dmrt-I* gene expression in the male conceptuses on day 9 [11]. *Dmrt-1* (also known as doublesex and mab-3 related transcription factor 1) is essential for the normal development of the mammalian testis [12]. More direct evidence of a carcinogenic role for OTA in the testis was provided by Mantle and Nolan [13]. These investigators administered 100 μg OTA as a daily dietary contaminant to 24 male Fischer rats for 35 weeks. Testis tumors developed in six of the rats (25%), an incidence rate equal to that of renal tumors in male Fischer rats chronically exposed to dietary OTA [14].

In adult rodents, OTA consumption causes adducts in liver, kidney and testicular DNA [15,16]. OTA also crosses the placenta and causes adducts in the liver and kidney DNA of newborns [17,18]. Because the testis and kidney develop from the same embryonic tissue, the mesonephros [19,20], we reasoned that OTA also may cause adducts transplacentally in the testis. We hypothesized that exposure to OTA during early life, either via consumption of foods that are contaminated with OTA or via exposure to OTA *in utero*, can cause lesions in testicular DNA. This research tests the hypothesis that *in utero* exposure to OTA causes adducts in testicular DNA and that these lesions are similar to those that can be produced in the kidney and testis by the consumption of OTA.

In addition, we showed previously that OTA forms a benzoquinone electrophile following activation by cytochrome P450 enzymes and radical species following activation of enzymes with peroxidase activities. These electrophiles react preferentially with deoxy guanine to form benzethenoadduct and C8 dG-OTA. Analysis of OTA-mediated DNA adduct by ^32^P post labelling method correlates with OTA chemistry and adduct spots derived from quinone electrophiles which are generated following activation by cytochrome P 450, while a C-8 dG OTA adduct is formed following activation of OTA by peroxidises, mainly contained in kidney and testis (for a review see [21]). In this study we further characterized the OTA metabolites in tissues from adult animals exposed to OTA.

## 2. Experimental Section

### 2.1. Chemicals

OTA (benzene free), 3'-dGMP, Proteinase K, RNase A, RNase T1 (boiled for 10 min at 100 °C to destroy DNases), and microccocal nuclease (dialyzed against deionized water) were purchased from Sigma-Aldrich (Saint-Quentin Fallavier, France); spleen phosphodiesterase (centrifuged before use) from Calbiochem (VWR, France); and nuclease P1 and T4 polynucleotide kinase from Roche (Meylan, France). [γ^32^P-ATP] (444 Tbq/mmol, 6000 Ci/mmol) was obtained from Amersham (Les Ullis, France); rotiphenol (pH 8) from Rothsichel (Lauterbourg, France); cellulose MN 301 from Macherey Nagel (Düren, Germany); polyethyleneimine (PEI) from Corcat (Virginia Chemicals, Portsmouth, VA); and Whatman No. 1 paper from VWR (France). Authentic 3’dGMP-OTA standard was prepared chemically prepared at Guelph University as described in detail [22]. All reagents, excluding solvents were of normal purity. Solvents were HPLC grade from ICS (Lapeyrouse-Fossat, France). Deionized water from a Milli-Q system (Millipore, France) was used for the preparation of all aqueous solutions for HPLC analysis.

4-S-OH-OTA, 4-R-OHOTA, and 10 hydroxylated OTA (10-OH-OTA) were a gift from Dr Størmer. Quinone OTA (OTHQ) was prepared as described in Gillman *et al*., 1999 [23]. Glutathione (GSH) metabolites such OTHQ-GSH and OTA-GSH were formed *in vitro* by autooxidation as follows: 10 µM of either OTHQ or OTA dissolved in phosphate buffer (100 mM, pH 7.4) were incubated for 45 min at 37 °C in presence of 1 mM GSH. N-acetylcystein (NAC) metabolites such OTHQ-NAC and OTA-NAC were formed *in vitro* by autooxidation as follows: 10 µM of either OTHQ or OTA dissolved in phosphate buffer (100 mM, pH 7.4) were incubated for 45 min at 37 °C in presence of 1 mM NAC.

### 2.2. Animals and Treatment

#### 2.2.1. Acute Gavage Feeding Studies

Seven week-old male BALB/c mice, weighing 20g ± 2, were purchased from IFFA-CREDO, L’Arbresle, France and were housed in individual cages and kept in environmentally controlled conditions (ventilation, 22 °C, 12 hours dark/light cycles). After an acclimation period of one week, 3 mice per group were given OTA dissolved in olive oil by a single intragastric intubation (oral gavage, 3.5; 7; 35; 70; 289; 578; 1056 µg/kg/b.w.). The control group of four mice received only olive oil. Mice were sacrificed 48 h after OTA administration. Testis and kidney were frozen immediately on dry ice and stored at −80°C pending analysis of OTA content, OTA metabolites content and DNA adducts. 

#### 2.2.2. Subchronic Feeding Studies

Seven week-old male BALB/c mice, weighing 20g ± 2, purchased from IFFA-CREDO, L’Arbresle, France were housed in individual cages and were kept under the same conditions as mice fed OTA by gavage. After an acclimation period of one week, 5 mice per group were given feed containing increasing amount of OTA (0.5, 1.4; 8; 20 µg/kg/b.w.) every day during 4 weeks. The feed (global rodent diets) was provided by Harlan Laboratory, Lyon, France. The feed was tested for the presence of mycotoxins after purification by partition for OTA, citrinin and aflatoxins and purification on IAC (Libios, France) for fumonisin B1 and zearalenone. The detection of the mycotoxins was performed by separation on HPLC with fluorimetric detection as described by Molinié *et al.*, 2005 [24] and by Nguyen *et al*., 2007 [25]). None of these mycotoxins were detected. Feed was experimentally contaminated with OTA by mixing it with a solution of OTA to reach the desired concentrations. Five animals received feed without OTA as control.

#### 2.2.3. Transplacental Studies

Six to 8 weeks old inbred Swiss (SWR/J) mice were purchased from The Jackson Laboratory (Bar Harbor, ME, USA). We first performed studies to determine the potential toxicity of OTA delivered i.p. to Swiss mouse dams at day 17 of gestation. Dams were monitored for signs of overt toxicity. Potential toxicity to offspring was measured by determination of number of pups born, average litter weight, survival to weaning and weight at 1 month. 

Animals were housed in plastic cages with hardwood shaving bedding. They were maintained on a 12 hours dark/light cycle and were allowed free access to food and water. After acclimation for two weeks, mice were mated by placing 2 to 3 females in a cage with one male. Pregnant mothers were treated on the 17th day of pregnancy (day one of pregnancy was the first day following overnight mating) by a high i.p. injection with either 2.5 mg/kg/b.w. OTA dissolved in olive oil or olive oil alone (control) at an equivalent volume. 

For adduct analysis, pups were sacrificed at birth, their gender was identified at necropsy, and kidneys and testes were removed from the males. Newborn testes and kidneys were frozen immediately following the same protocol as for the gavage-fed mice. Additionally, male mice from 4 litters (two OTA-treated, two controls) were maintained for one month and then sacrificed. Kidney and testicular tissues were removed, frozen, and used for quantitation of DNA adducts.

### 2.3. OTA and OTA Metabolites Determination in Tissues

#### 2.3.1. Analysis of OTA Content in Tissues of Adult Mice

Tissue OTA content was extracted and measured as described by Petkova-Bocharova *et al.*, 2003 [26]. Briefly, 500 mg of tissue was homogenized with 10 mL of 0.1 M MgCl_2_/0.05 M HCl, pH 1.5 and extracted three times with chloroform. Combined chloroform extracts were extracted twice against sodium hydrogen carbonate, the aqueous phase acidified to pH 1.5, and further extracted with chloroform. Combined chloroform extracts were dried under vacuum, dissolved in methanol, filtered, dried under nitrogen and dissolved in 200 µL of methanol for HPLC analysis. OTA was analysed by reverse phase HPLC using a Nucleosil 100-3-C18 column, 15 cm under isocratic elution (Methanol, 600 mL; acetonitrile, 600 mL; ultrapure water, 800 mL; sodium acetate, 3 H_2_O, 0.68 g; glacial acetic acid, 28 mL) run 30 minutes. Detection was performed with a programmable fluorimeter GTI spectrovision (excitation 340 nm, emission, 465 nm). HPLC grade solvent columns were supplied by ICS (Lapeyrouse-Fossat, France). The limit of detection (LOD) was 0.05 ng/g; the limit of quantification (LOQ) 0.2 ng/g.

#### 2.3.2. Analysis of OTA Metabolites in Tissues of Adult Mice

The metabolites were separated on prontosil 250 mm × 4 mm, 3 µ using the following gradient: solvent A: MeOH/ACN/6.5 mM ammonium formate (200/200/600) adjusted to pH 3 with formic acid; solvent B: MeOH/ACN/6.5 mM ammonium formate (350/350/300) adjusted to pH 3 with formic acid. Program: T0 min 100% A; T10 min 100% A; T25 min 30% A; T30 min 30% A; T45 min 0% A; T55 min 0% A; T58 min as described in Faucet-Marquis *et al*., 2006 [27]. Detection was performed with a programmable fluorimeter GTI spectrovision (ex = 350 and em = 510 nm), allowing better detection of some OTA metabolites [28].

### 2.4. DNA Adduct Detection and Identification of C-C8dG OTA Adduct

Kidney and testis DNA isolation and purification as well as the method used for ^32^P-postlabeling were performed as described by Faucet *et al.*, [22] and in detail by Pfohl-Leszkowicz & Castegnaro [29]. In brief, the equivalent of 7 μg of DNA was dried *in vacuo*, dissolved in 10 μL of the mix containing 1 μL of micrococcal nuclease (2 mg/mL corresponding to 500 U), spleen phosphodiesterase (15 mU/μg DNA), 1 μL of sodium succinate (200 mM), and 1 μL of calcium chloride (100 mM, pH 6) and digested at 37 °C for 4 h. The digested DNA was then treated with 5 μL of the mix containing 1.5 μL of nuclease P1 (4 mg/mL), 1.6 μL of ZnCl_2_ (1 mM), and 1.6 μL of sodium acetate (0.5 M, pH 5) at 37 °C for 45 min. The reaction was stopped by the addition of 3 μL of Tris base 500 mM. The DNA adducts were labeled as follows: to the nuclease P1 digest, 5 μL of the reaction mixture containing 2 μL of bicine buffer [bicine (800 μM), dithiothreitol (400 mM), MgCl_2_ (400 mM), and spermidine (400 mM) adjusted to pH 9.8 with NaOH], 10 U of polynucleotide kinase T4, and 100 μCi of [γ-^32^P]ATP (specific activity 6000 Ci/mmol) was added and the mixture incubated at 37 °C for 45 min. Normal nucleotides, pyrophosphate, and excess ATP were removed by chromatography on PEI/cellulose TLC plates (D1) in 2.3 M NaH_2_PO_4_ buffer, pH 5.7, overnight. The original (2 cm) areas containing labeled adducted nucleotides were cut out and transferred to another PEI/cellulose TLC plate, which was run (D2) in 4.8 M lithium formate and 7.7 M urea, pH 3.5, for 3 h. A further (D3) migration was performed after turning the plate 90° anticlockwise in 0.6 M NaH2PO4 and 5.95 M urea, pH 6.4, for 3 h. Finally, the chromatogram was washed in the same direction in 1.7 M NaH2PO4, pH 6, for 2 h (D4). Adduct profiles were analyzed qualitatively and semi-quantitatively by autoradiography of the plates, carried out at −80 °C for 48 h in the presence of an intensifying screen using a radioanalytical system of image analysis (AMBIS, Lablogic). 

Identification of one DNA adduct was made by comigration with authentic standard. The standard OTA-3’P-dGMP adduct (C-C8) was dissolved in sterile deionised water. A dilution corresponding to 143 fmol of adducts was ^32^P-postlabeled. OTA adduct in testis DNA was identified by either co-chromatography of DNA adducts from the testis (3.5 µg of digested DNA) and OTA standard or with DNA adducts from kidneys (3.5 µg of digested DNA in the conditions used for purified DNA). Isolation of this specific adduct was performed following the technique developed by Mantle *et al.*, 2010 [30]. In brief, the cellulose in the central area corresponding to spots of interest was scraped off. The cellulose was suspended in methanol, shaken and centrifuged; the solution and the extraction cycle was repeated. Residual cellulose was then similarly extracted with triethanolamine. Combined extracts were evaporated to dryness in vacuum. The residue was solved in methanol and put on PEI cellulose for additional D2, D3, D4.

## 3. Results

### 3.1. Acute and Subchronic Effect of OTA on Adult Male Mice

Three animals per group received a single dose of OTA and were sacrificed 48 h after treatment. Three control animals received only vehicle. The control animals also were sacrificed 48 h later. For the subchronic study, five animals per group were fed OTA (in feed). DNA was extracted from kidney and testis. OTA and its derivatives were extracted and analyzed by HPLC.

#### 3.1.1. DNA Adduct Detection

**Figure 1:**
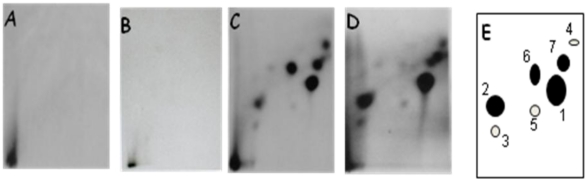
DNA adduct pattern in tissues of mice treated with Ochratoxin A. (A) kidney DNA from control mice; (B) testis DNA from control mice; (C) kidney DNA adduct from mice treated with 1056 mg/kg b.w.; (D) testis DNA adduct from mice treated with 1,056 mg/kg b.w.; (E) Scheme of numbering of the individual DNA adducts. Exposure time of the film was 48 h.

DNA adducts were analyzed in kidney and testes of male mice treated either acutely or subchronically by increasing amount of OTA using the postlabeling technique after nuclease P1 enrichment. This technique allows detection of covalent DNA adducts only. No DNA adducts were observed either in kidney and testis of mice receiving only vehicle. Several individual DNA adducts are observed in kidney and testis after exposure over 7 µg/kg b.w. An example of DNA adduct patterns is shown in Figure 1.

**Figure 2:**
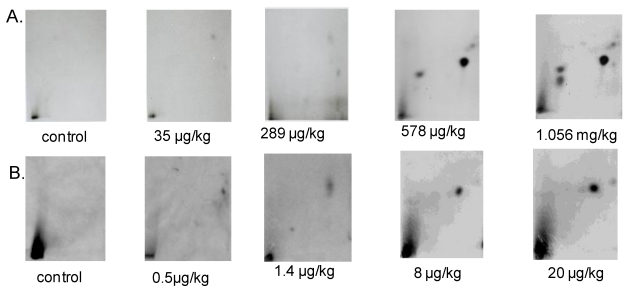
DNA adduct pattern in testis of male mice exposed to ochratoxin A. (A) acute exposure (gavage); (B) mice were fed 4 weeks with feed containing OTA. Doses are expressed as µg/kg b.w. The films were exposed for 24 h panel (A) and 48 h panel (B).

The numbers of individual adducts and the intensity of the DNA adducts increased with the exposure. A low exposure led mainly to the formation of the DNA adducts 1 and 2. High acute exposure induced at least 7 different DNA adducts (Figure 2 A). The DNA adducts formed in testis are similar to those formed in kidney (Figure 1C & Figure 1D). DNA adduct 1 corresponding to C-C8dG OTA (see below 3.2.2 and Faucet *et al*., 2004 [22]; Mantle *et al.*, 2010 [30]) is the main adduct in both organs. This adduct is also formed even after chronic exposure as low as 0.5 µg/kg b.w, and increased with dose (Figure 2B). Although adduct 6 is formed in both organs, it is formed to larger extent in kidney after acute exposure. After chronic exposure, adducts 2, 3 and 7 are also formed in testis.

**Figure 3:**
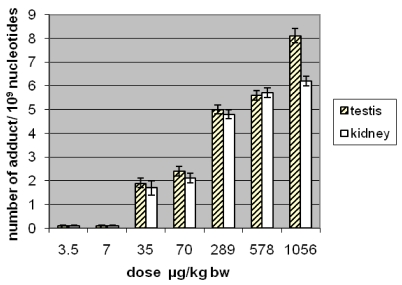
Amount of C-C8dG OTA, expressed as relative adduct level, in organs of mice exposed to increasing amount of ochratoxin A (single dose). Amount in testis = hatched bar; amount in kidney = white bar.

The amounts of C-C8dG OTA observed in kidney and testis of mice exposed by gavage are given in Figure 3. The amount of C-C8 dG-OTA adduct (spot labeled number 1 in the scheme Figure 1) formed in kidney and testis are in the same range for dose treatment below 1056 µg/kg b.w. In the case of high exposure, the amount of C-C8dG OTA was 25% higher in testis than in kidney.

#### 3.1.2. Amount of OTA in Kidney and Testis of Adult Mice

Table 1 summarizes the OTA content in testis and kidney from BALB/c mice, administered increasing doses of OTA by a single gavage. The data show that the quantity of OTA stored in tissues increased with the dose administered, but the increase was not linear. The kidneys of male mice accumulated more OTA than did the testes at all doses. An increase in OTA administered over the low dose of 3.5 µg/kg b.w. by 300-fold led to an approximately 100-fold increase in OTA content in kidney. In the testis, OTA only accumulated when the dose was higher than 35 µg/kg b.w. (corresponding to 0.2 ppm in the feed). Following treatment of mice by a 15-fold higher amount of OTA (1,056 µg/kg b.w. *vs.* 70 µg/kg b.w.), the OTA in testis was 30-fold higher.

**Table 1:** OTA content in kidney and testis of BALB/c treated with increasing concentrations of OTA by oral gavage

OTA administered by gavage µg/kg b.w.	OTA content in testis* ng/g	OTA content in kidney* ng/g
0	nd**	nd
3.5	nd	3.5 ± 1.4*
7	nd	8.2 ± 0.7
35	nd	110 ± 2.5
70	1.6 ± 0.2	123 ± 2
289	3 ± 0.1	110 ± 27
578	9.6 ± 1.5	210 ± 30
1056	50 ± 15	330 ± 60

* average of three animals; ** not detectable (below limit of detection)

#### 3.1.3. Identification of OTA Metabolites in Tissues

**Figure 4:**
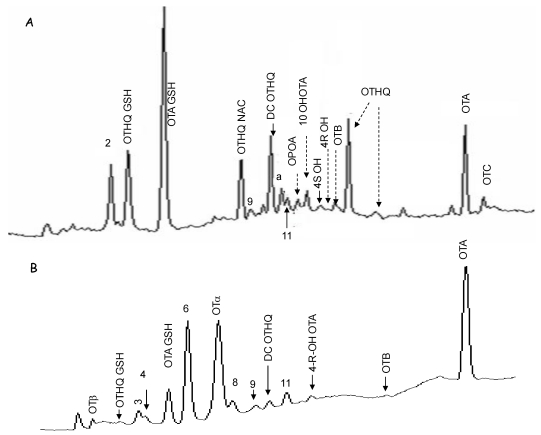
OTA metabolites detected in urine (A) and liver (B) of OTA treated males OTHQ (quinone OTA); GSH conjugated to glutathione; NAC conjugated to *N*-acetylcystein, DC OTHQ decarboxylated OTHQ; OP OA open ring OTA; OTB dechlorinated OTA; OTC ethylated OTA.

OTA and its metabolites were extracted from urine, liver, kidney and testis of mice fed increasing amount of OTA for 4 weeks. The metabolites were separated by gradient elution. An example of separation is given in Figure 4. Most of the OTA metabolites have been identified by LC MS/MS [27]).

In male kidney and in testis, twelve metabolites were formed in both organs: OTβ; metabolite “2”; OTHQ-GSH; OTA-GSH; OTα; OTHQ-NAC; DC-OTHQ; 4R-OH-OTA; OTHQ; OTC. Two metabolites were observed in kidney but not in testis: 4S-OH-OTA and metabolite “9”. Some OTA metabolites were formed in liver but not in kidney and testis, such metabolites 3, 4, 6, 11 and OTB. The OTHQ metabolites (OTHQ; DC-OTHQ, OTHQ-NAC) are not detected in liver.

### 3.2. Effect of Transplacental OTA Contamination on Male Mice Pup

#### 3.2.1. Transplacental Toxicity

**Figure 5:**
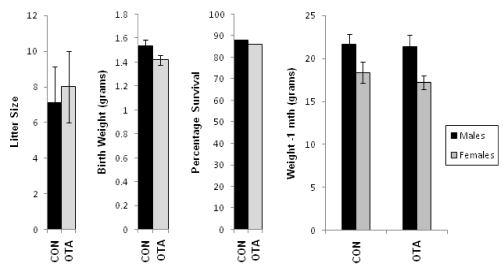
Comparison of litter size, survival and weight of mice treated or not by OTA (CON = control animal; OTA = ochratoxin A treated animal).

No overt toxicity of OTA was detected in either dams or litters. An average of 7–8 pups were delivered to both control and OTA-treated mothers, with an average birth weight of 1.5 g/pup and 1.4 g/pup, respectively. Survival to weaning was also equivalent (50/57 in controls *vs.* 48/56 in OTA-treated) as was weight at 1 month of age (21.7 g/male and 18.4 g/female in controls *vs.* 21.4 g/male and 18.2 g/female in OTA-treated) (Figure 5).

#### 3.2.2. DNA Adduct Analysis in Kidney and Testis of Male Mice Pup

**Figure 6:**
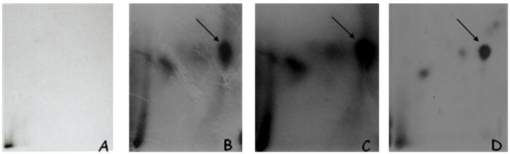
DNA adduct pattern of testis of one-month old mice. (A) litter from untreated mother mice; (B, C, D) litter from OTA-treated mother (arrow points C-C8dG OTA); (B & C) correspond to two animals from Mother Number 2; (D) corresponds to one animal from Mother Number 1.

DNA adducts were analyzed in kidney and testis from newborn male littermates exposed to OTA at 17 days gestation and from 8 male mice aged 1 month. Three male mice were born from one mother and five from another. No adducts were detected in the testis and kidney of untreated mice. Several adducts were observed as well in kidney as in testis of pups born from OTA-treated mothers. A representative example of DNA adduct pattern in testis is shown in Figure 6.

**Figure 7:**
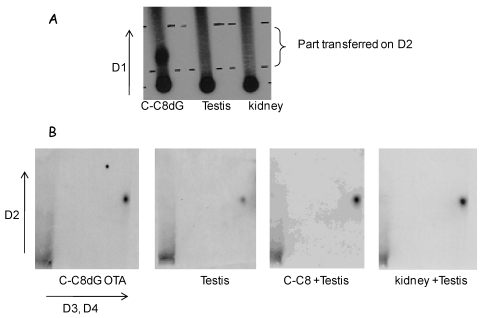
Comigration of mouse testis DNA with kidney DNA or C-C8 dG OTA standard (A) D1 migration; The upper part is cut out and transferred onto new plates as described in Mantle *et al.*, 2010 [30]; (B) C-C8dG OTA spot; Testis spot; comigration ½ C-C8dGOTA + ½ Testis; comigration ½ kidney + ½ testis.

**Figure 8:**
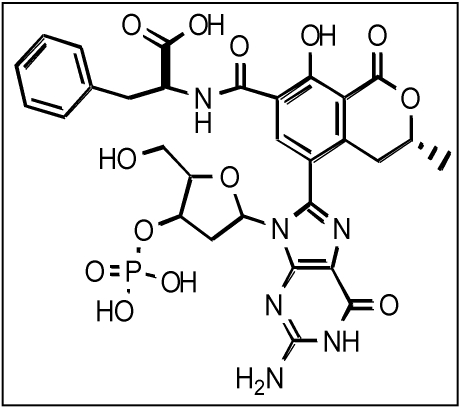
Structure of C-C8 dG-OTA.

Mainly, three adducts were formed in kidney and testis of the male pups. These adducts correspond to adducts numbered “2”, “6”, “1” observed in adult mice (see Figure 1 for numbering). The intensity of the adducts differed according to the tissue and age of the animals. In newborn male mice adduct “6” is formed neither in kidney nor in testis. This suggest that adduct “6” likely results from OTA contamination via suckling, and explains why the DNA adduct amounts are higher in one month pups than in newborns. In order to confirm the structure of the adduct 1, comigration of testis DNA was performed with either kidney DNA or the C-C8 dGMP-OTA DNA standard. After D1 migration, the spot corresponding to C-C8dG OTA was cut out in each sample (Figure 7A). These spots were transferred onto a new plate and eluted in two dimensions. The main spot in kidney co-migrated with the main spot in testis. This spot co-migrated with C-C8 dGMP-OTA (Figure 7B). The structure of this adduct is given Figure 8.

The mean C-C8dGMP-adduct level, measured on the organs in newborns, was 5.2 ± 0.1 and 4.2 ± 0.2 nucleotides per 10^9^ nucleotides in testis and kidney, respectively. The quantity of this latter adduct for each one-month old animal is shown in Table 2. 

**Table 2:** Amount of C-C8dG OTA in kidney and in testis of one-month old mice contaminated with OTA via the transplacental route.

	Animal Numbering	C-C8dG OTA in Testis	C-C8dG OTA in Kidney
Mother 1	1	5.5 ± 0.3	4.8 ± 0.2
	2	5.7 ± 0.3	5.1 ± 0.3
	3	5.1 ± 0.3	4.7 ± 0.2
Mother 2	4	7.3 ± 0.5	6.3 ± 0.2
	5	8.3 ± 0.5	6.5 ± 0.4
	6	7.7 ± 0.3	6.9 ± 0.5
	7	7 ± 0.6	6.6 ± 0.3
	8	7.8 ± 0.4	6.8 ± 0.5

The amount of C-C8dG OTA was significantly higher in testis compared to kidney. Pups from the same mother have a similar amount of C-C8 dG OTA.

## 4. Discussion

Our results from gavage-feeding experiments are similar to those described previously [15,16]. Unlike Gharbi *et al.*, [17], who administered 289 µg/kg b.w. OTA every 48 hrs for a period of 8 weeks (*i.e.*, chronic administration), we showed that OTA induced adducts in testicular DNA after a single (acute) exposure using OTA doses that were 10-fold lower (<1 ppm in food). The number of individuals adducts increased with the dose. Daily intake of low amounts of OTA in feed induced the formation of the main adduct. 

To our knowledge, this is the first study to demonstrate that maternal exposure to OTA causes DNA adducts in the testis of newborn mice. These adducts are chromatographically similar to adducts in kidney and in testicular DNA that were produced by feeding OTA to adult mice by gavage. The main adduct observed in the testis comigrated with the C-C8dGMP OTA adduct which was also the main adduct in the kidney and has been recently identified by MS/MS (Mantle *et al.*, 2010 [30]). This adduct is dechlorinated and thus could be formed only after OTA biotransformation [21,22,27,29,30]. The susceptibility of male rats to kidney cancer after OTA exposure is highly dependent on enzymes regulated by sexual hormones such CYP 2C [28] but also on enzymes involved in oxidative pathways [21] such cyclooxygenases [32,33,34] and glutathione conjugation [27,35]. Interestingly, Verma & Chakraborty [36] observed a decrease of catalase, SOD, glutathione peroxidase activities and of the amount of glutathione in testis of mice exposed to OTA. In testis and in kidney of mice fed OTA, we detected OTHQ formed via the peroxidase pathway [21]. In testis and kidney, several quinone metabolites were formed (OTHQ = ochratoxine hydroquinone; DC-OTHQ = decarboxylated OTHQ; OTHQ-GSH = conjugated to glutathione; OTHQ-NAC = conjugated to N-acetylcystein). These metabolites were not found in liver. This indicates that quinone formation arose in tissues having cylooxygenase, CYP 2C and glutathione conjugation pathways as it is the case mainly in kidney and testis. Interestingly, CYP 2C is induced by sex hormones [38] and is regulated by CYP 3A4 [38] which is abundantly expressed in the human liver, intestine and kidney. The CYP 3A5*1 allele was more prevalent in patients suffering from Balkan endemic nephropathy (BEN), for which OTA is a plausible etiologic agent, with a frequency of 9.4% *versus* 5.4% in controls, and was associated with a higher risk for BEN (Odds Ratio = 2.41) [39].

Adduct levels appeared to increase over time, as levels in month-old mice were greater than in newborns. This could be explained by the high affinity of OTA for proteins, accumulated in tissue of newborn and released for a long time after exposure. In addition, OTA is excreted in milk and thus exposure of the newborn to OTA continues after birth (for a review see [2]).

The C-C8dGMP OTA adduct is the same adduct consistently observed in kidney tumors from patients with BEN in the Balkans and in other European countries [40,41,42]. In this regard, it is noteworthy that a recent epidemiologic study of BEN in the Vratza District, Bulgaria (where BEN was first characterized), found that cases of BEN in adults were associated with the presence of BEN in the mother, but not in the father [43]. These findings suggest that exposure to OTA *in utero* may play an etiologic role in BEN. 

Our finding that OTA caused DNA adducts in testes of newborns is consistent with the hypothesis of a possible role for exposure to OTA in testicular cancer [10]. Little is known about the etiology of testicular cancer, but its peculiar age-incidence curve, showing a peak in adolescence, and the identification of carcinoma *in situ* of the testis in pre-pubertal testes, suggests that the causes of testicular cancer occur *in utero* or peri-natally [44,45]. The only established cause of testicular cancer is cryptorchidism (failure of the testes to descend into the scrotum). However, it is widely believed that it is not cryptorchidism *per se* that causes testicular cancer, but rather, that cryptorchidism and testicular cancer share a common cause. A role for endocrine disruptors has long been suggested in testicular cancer, male infertility and in cryptorchidism (*i.e.*, the “testicular dysgenesis syndrome”) but the identity of the agent(s) responsible for these epidemics is a mystery [46]. It is intriguing in this regard that OTA is known to cause inhibition of testosterone secretion [47], poor semen quality [48,49,50] and cryptorchidism. Thus, Wangikar *et al*., [51] found a significantly increased incidence of cryptorchid testes (2/12 OTA-treated pups *vs.* 0/43 controls) in Wistar rats given 0.75 mg OTA/kg on days 6–15 of gestation (P = 0.006). Similarly, Patil *et al*., [52] studied the effects of a single dose of OTAat 2.75 mg/kg body weight on maternal and fetal toxicity of pregnant Wistar rats. They observed a high incidence of gross abnormalities to fetuses from OTA exposure on gestation day (GD) 6 and 7 (13 and 18%, respectively). Five instances of cryptorchid testes (of 92 foetuses examined) were observed on GD 6–10 and GD 13. 

The recent observation that *Dmrt-1* gene expression is markedly reduced in conceptuses of male mice from dams treated with OTA is particularly noteworthy [11]. Although *Dmrt-1* is essential for the embryonic development of the testis in humans, *Dmrt-1* is involved in the postnatal development of the testis in rodents [53]. This may explain the development of testis cancer in older Fischer rats chronically exposed to OTA via the diet [13].

## 5. Conclusions

We demonstrated that intrauterine exposure to OTA produces DNA adducts in the testes of newborn mice and that these adducts are similar to the DNA adducts that are observed in the kidney and testis of adult mice that are exposed to OTA via the diet. These data add additional support to the hypothesis that OTA may play a role in the etiology of testicular cancer [10]. 
